# Ultra-high-performance liquid chromatography high-resolution mass spectrometry–based screening of purine and pyrimidine metabolites in cow milk collected across 4 different seasons

**DOI:** 10.3168/jdsc.2025-0924

**Published:** 2026-03-12

**Authors:** Michela Errico, Gabriele Rocchetti, Marco Lapris, Valentina Novara, Paolo Bellassi, Francesca Fumagalli, Annamaria Boldini, Angelo Stroppa, Antonio Gallo

**Affiliations:** 1 Department of Animal Science, Food and Nutrition, Università Cattolica del Sacro Cuore, Piacenza, 29122, Italy; 2 Department for Sustainable Food Process, Università Cattolica del Sacro Cuore, Piacenza, 29122, Italy; 3 Consorzio Tutela Grana Padano, 25015, San Martino Della Battaglia, Desenzano del Garda, BS, Italy

## Abstract

Milk nucleotides have received little attention compared with other metabolite classes, despite their central role in rumen microbial activity and potential value as markers of nutritional and environmental dynamics. This study applied untargeted metabolomics to screen purine- and pyrimidine-related metabolites in bulk tank milk collected from dairy herds in the Po Valley (Italy) across 4 seasons under a standardized corn silage-based total mixed ration. A total of 82 metabolites were annotated, with purine and pyrimidine metabolism emerging as central hubs in the milk metabolome and showing connections with amino acid and nitrogen pathways. Multivariate analyses revealed that summer and fall samples had more distinctive nucleotide profiles than spring and winter, with adenosine- and guanosine-related compounds being the main discriminants. Phosphoribosyl pyrophosphate was identified as a key intermediate linking purine and pyrimidine metabolism, whereas carbamoyl phosphate accumulation in summer highlighted the sensitivity of pyrimidine pathways to environmental stressors. Semiquantitative evaluation of known markers showed stable levels of uric acid and allantoin across seasons, whereas β-aminoisobutyric acid, a pyrimidine catabolite, increased in summer. These findings suggest that both pyrimidine and purine metabolism are responsive to seasonal influences under controlled feeding, positioning β-aminoisobutyric acid, and potentially carbamoyl phosphate, as candidate tracers of summer-related metabolic adaptations in dairy cows.

## Highlights

• Milk nucleotides were systematically profiled across 4 seasons.
• Purine and pyrimidine metabolism emerged as central metabolic pathways.
• Summer and fall milk showed the most distinctive nucleotide profiles.
• β-Aminoisobutyric acid increased in summer as a potential biomarker.
• Carbamoyl phosphate suggested links between heat stress and pyrimidine turnover.

## Graphical Abstract

**Figure:**
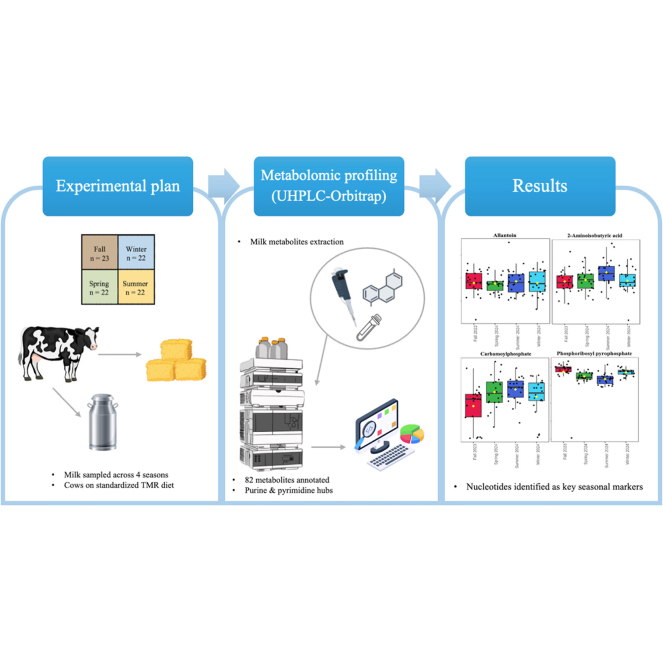
**Summary:**This study explored seasonal variations in milk nucleotide composition using untargeted metabolomics. Bulk-tank milk was collected from dairy herds in the Po Valley (Italy) across 4 seasons under a standardized corn silage-based diet. Ultra-high-performance liquid chromatography-high-resolution mass spectrometry (UHPLC-HRMS) profiling enabled the putative annotation of 82 nucleotide-related metabolites, with purine and pyrimidine pathways emerging as central hubs of the milk metabolome. Multivariate models revealed that summer and fall milk exhibited more distinctive nucleotide signatures compared with spring and winter. Among the discriminant metabolites, β-aminoisobutyric acid (a pyrimidine catabolite) increased significantly in summer, whereas carbamoyl phosphate suggested links between heat stress, nitrogen balance, and pyrimidine metabolism. In contrast, allantoin and uric acid remained stable across seasons, reflecting controlled feeding. These findings demonstrate that milk nucleotide metabolism is sensitive to environmental influences, with selected compounds acting as potential biochemical tracers of seasonal adaptation in dairy cows. TMR = total mixed ration.

Milk is a complex biological fluid whose composition reflects not only the physiology of the dairy cow, but also the influence of diet, environment, and rumen microbial activity (Zhu et al., 2021; Suh, 2022). In recent years, metabolomics has been increasingly applied to characterize milk and to identify metabolic signatures related to nutrition, health, and product quality (Rocchetti and O'Callaghan, 2021). Particular attention has been devoted to amino acids, lipids, and organic acids, whereas nucleobases and their derivatives have received little or no attention (Rocchetti et al., 2022; Giagnoni et al., 2025). Purine and pyrimidine metabolites play a central role in microbial metabolism, originating from nucleic acid degradation, and are widely recognized as tracers of rumen microbial protein synthesis (Schager et al., 2003; Stentoft et al., 2015). Dietary factors have been shown to modulate purine and pyrimidine levels in rumen fluid and plasma, suggesting that these compounds may serve as indicators of nutritional and metabolic dynamics in dairy cows (Stentoft et al., 2014). Stentoft et al. (2014) developed an HPLC tandem MS (**MS/MS**) approach to quantify purine and pyrimidine bases, nucleosides, and degradation products in bovine plasma; however, to date, no studies have investigated their presence and seasonal variation in bovine milk using a high-resolution MS approach. The Po Valley (Northern Italy) is a major dairy region and the main source of milk for Protected Designation of Origin (**PDO**) hard cheeses. Farms typically adopt a TMR where corn silage is the main forage, often combined with grass or legume silages (Gallo et al., 2022; Rocchetti et al., 2022). Seasonal factors such as temperature, humidity, and management may affect rumen fermentation and, indirectly, milk composition, even under standardized feeding (Islam et al., 2021). Therefore, profiling purine and pyrimidine metabolites in milk across seasons offers a novel way to explore their potential as biochemical indicators of microbial activity and environmental adaptation. The objective of this study was to perform an untargeted ultra-HPLC high-resolution MS (**UHPLC-HRMS**) screening of purine and pyrimidine metabolites in cow milk sampled across 4 seasons under a defined PDO feeding regimen. The milk samples were collected from commercial dairy farms located in the Po Valley area (Northern Italy), all belonging to the Grana Padano PDO production district and rearing Holstein dairy cows. All herds were fed a standardized TMR based on corn silage as the main forage ingredient. Representative TMR samples were collected in each season and analyzed by near-infrared spectroscopy, confirming a comparable composition. In particular, the PDO-based feeding constraints resulted in limited variability of feedstuffs and nutrient composition across seasons, allowing seasonal environmental effects to be evaluated under comparable dietary conditions. On a DM basis, the average TMR composition considering the 4 seasons was as follows: 14.9% crude protein, 4.1% ether extract, 16.1% fiber, 6.1% ash, 26.5% starch, 32.4% NDF, 18.2% ADF, 2.6% ADL, 5.3% soluble protein, and 5.2% sugars, reflecting a herd-level diet representative of commercial dairy systems with cows at different stages of lactation and productive performance, rather than a diet specifically formulated for high-producing dairy cows. The mean inclusion rate of corn silage in the TMR was 26.2% DM, with limited seasonal variation (27.7% in fall 2023, 27.1% in winter 2024, 25.9% in spring 2024, and 20.6% in summer 2024), confirming the standardized feeding regimen adopted across all herds. At the herd level, DMI was calculated as described by Gallo et al. (2022), corrected for DM content (Atzori et al., 2021). Mean DMI values were 25.84, 25.53, 27.45, and 26.30 kg/cow per day in fall, winter, spring, and summer, respectively. Composite bulk tank milk samples were collected in summer (n = 22), fall (n = 23), winter (n = 22), and spring (n = 22), for a total of 89 samples, and were transported under refrigeration, aliquoted, and stored at −20°C until analysis. Environmental data were obtained from the Regional Environmental Protection Agency (ARPA) of Lombardy and Emilia-Romagna (Italy) using meteorological stations located nearest to each herd based on geographic coordinates. The daily minimum, maximum, and average temperature (**T_min_**, **T_max_**, **T_avg_**) and humidity (**H_min_**, **H_max_**, **H_avg_**) were recorded, and the temperature-humidity index (**THI**) was calculated as previously described (Del Corvo et al., 2021). Milk metabolites were extracted following Rocchetti et al. (2022) with minor modifications. Briefly, skim milk obtained by centrifugation (4,000 × *g*, 10 min, 4°C) was extracted with cold acetonitrile (3% formic acid), vortexed, sonicated, centrifuged (18,000 × *g*, 10 min, 4°C), incubated overnight at −18°C, and filtered (0.22 μm) prior to UHPLC-HRMS analysis. Untargeted metabolomic profiling was performed using a Q-Exactive Focus Orbitrap mass spectrometer coupled to a Vanquish UHPLC system (Thermo Fisher Scientific), as previously reported (Rocchetti et al., 2022). Data were processed using MS-DIAL (v. 4.90; https://systemsomicslab.github.io/compms/msdial/main.html) and annotated against the Bovine Metabolome Database (Foroutan et al., 2020), applying mass tolerances of 0.05 and 0.1 Da for MS^1^ and MS/MS, respectively, and a 5-ppm accuracy threshold. Purine and pyrimidine metabolites were specifically curated, with β-aminoisobutyric acid (**BAIBA**), allantoin, and uric acid semi-quantified using authentic standards (Giagnoni et al., 2025). Normalized metabolite intensities were used for comparative analyses. Multivariate analyses were performed in MetaboAnalyst 6.0 (Pang et al., 2024) on median-centered, Pareto-scaled, and log_10_-transformed data, using hierarchical cluster analysis and partial least squares discriminant analysis (**PLS-DA**; 5-fold cross-validation; R^2^ and Q^2^ >0.5). Discriminant metabolites were identified by variable importance in projection (**VIP**) scores (>0.8) and volcano plots (fold-change >1.2; ANOVA *P* < 0.05). Random forest (**RF**) classification (500 trees) evaluated model performance using out-of-bag error rates. Semiquantitative data were further analyzed by one-way ANOVA followed by Duncan's post hoc test (*P* < 0.05). Pathway and metabolite set enrichment analyses were conducted using the *Bos taurus* Kyoto Encyclopedia of Genes and Genomes library. Overall, 82 metabolites were annotated (supplemental dataset, see Notes), with 25 compounds associated with pyrimidine degradation pathways, including BAIBA, dihydrothymine, thymine, alanine, uracil, and cytosine, whereas 21 compounds were assigned to purine degradation pathways, such as allantoin, uric acid, adenosine, and guanosine. Among the most abundant milk metabolites, guanosine emerged as a predominant nucleoside reflecting nucleotide turnover and microbial activity, consistent with its role in energy transfer and signaling (Mancinelli et al., 2020). β-Aminoisobutyric acid, a thymine catabolite, was also abundant, indicating active pyrimidine degradation and potential links to metabolic adaptation (Rocchetti et al., 2022). Additionally, 5-aminoimidazole ribonucleotide, a key intermediate in purine and thiamine biosynthesis (Caspi et al., 2014), and cytidine, a pyrimidine nucleoside involved in RNA and phospholipid synthesis (Tiemeyer et al., 1984), were detected, supporting the contribution of nucleotide metabolism to the milk metabolome. To evaluate seasonal effects, unsupervised hierarchical clustering (Figure 1A) revealed 2 main groups, with spring and summer samples clustering together and winter and fall showing similar profiles. Pathway-based network analysis (Figure 1B) confirmed purine and pyrimidine metabolism as central hubs, closely connected to nucleotide turnover, energy balance, and microbial activity, reflecting the interplay between rumen-derived precursors and mammary metabolism. Secondary links with amino acid, nitrogen, pantothenate/CoA, sulfur, and one-carbon metabolism further highlight the integration of nucleotide pathways with broader cellular functions. Overall, this network overview indicates that milk contains a complex metabolite repertoire extending beyond classical energy substrates and amino acids to include nucleotide-derived intermediates with potential biomarker value. Based on this overview, supervised multivariate analysis was applied to assess whether purine- and pyrimidine-related metabolites captured seasonal dynamics under controlled feeding conditions. The PLS-DA biplot (Figure 2A) showed clear separation of summer and fall samples, whereas spring and winter partially overlapped, resulting in moderate model performance (Q^2^ < 0.5; R^2^ < 0.8; cumulative value = 0.59 over 5 components). This pattern suggests that periods characterized by more extreme environmental conditions generate more distinctive nucleotide profiles. Several nucleotides and nucleosides, including deoxyadenosine triphosphate (**dATP**), ATP-related compounds, and guanosine derivatives, contributed strongly to seasonal discrimination. Variable importance analysis identified 18 key metabolites, with dATP, adenosine thiamine triphosphate (**AThTP**), guanosine 2',3′-cyclic phosphate (**2′,3′-cGMP**), and diadenosine heptaphosphate (**AP7A**) showing the highest VIP scores (>2.2). Fall samples were characterized by increased levels of dATP, AThTP, AP7A, B-AMP, 5′-deoxyadenosine, xanthosine, guanosine, ADP, and deoxycytidine monophosphate, whereas guanosine tetraphosphate adenosine was most strongly associated with spring. In summer, 2′,3′-cGMP, 8-hydroxyguanosine, and carbamoyl phosphate emerged as the main discriminants, whereas winter samples showed higher levels of uridine diphosphate acetylgalactosamine 4-sulfate (**UDP-galNAc-S**), deoxycytidine diphosphate, deoxyinosine monophosphate, deoxycytidine triphosphate, and 7-methyladenine (Figure 2A). Overall, many discriminant metabolites were directly linked to nucleic acid turnover and microbial activity, supporting the hypothesis that seasonal changes in rumen fermentation are reflected in milk nucleotide metabolism. Some discriminant metabolites (e.g., carbamoyl phosphate and UDP-galNAc-S) were linked to broader nitrogen and carbohydrate metabolism, indicating that seasonal effects on milk composition extend beyond purine and pyrimidine catabolism alone. In particular, the increased abundance of carbamoyl phosphate in summer may reflect an adaptive shift at the interface between nitrogen metabolism and nucleotide biosynthesis, as this metabolite represents a key intermediate connecting the urea cycle with de novo pyrimidine synthesis (Watford, 2003). Under heat stress, dairy cows typically experience reduced DMI values, altered nitrogen balance, and increased protein turnover (Ríus, 2019; Chen et al., 2024), which may impair urea cycle efficiency and redirect carbamoyl phosphate toward pyrimidine metabolism. Although mechanistic conclusions cannot be drawn, the seasonal accumulation of carbamoyl phosphate supports the view that pyrimidine metabolism is particularly sensitive to environmental conditions and that nucleotide-related metabolites may serve as tracers of physiological adaptation in dairy cows. Environmental data provided additional context for these findings. Average THI values indicated thermoneutral conditions in fall, winter, and spring (48.8, 51.9, and 62.2, respectively) and moderate to severe heat stress in summer (78.1). These seasonal patterns mirrored the metabolomic differentiation observed in milk, with the highest THI coinciding with increased carbamoyl phosphate and BAIBA. This correspondence supports the hypothesis that pyrimidine metabolism responds sensitively to environmental heat load, even under standardized feeding conditions. Random forest classification further assessed the seasonal discriminative power of purine and pyrimidine metabolites (Figure 2B). The overall out-of-bag error rate (0.416) indicated moderate classification performance, with lower misclassification rates for summer and fall (0.182 and 0.130), suggesting more distinctive nucleotide profiles during periods of stronger environmental contrast. In contrast, spring and winter showed higher misclassification rates (0.773 and 0.591), likely reflecting the transitional nature of spring conditions in the Po Valley and partial overlap with adjacent seasons. Overall, RF confirmed the trends observed by PLS-DA, indicating that seasonal effects on milk nucleotide composition are more evident under specific climatic conditions. Notably, most discriminant features were adenosine- and guanosine-related compounds, consistent with the central role of adenine and guanine nucleotides in energy transfer, signaling, and nucleic acid turnover (Mancinelli et al., 2020; Popović et al., 2024). Their seasonal variability likely reflects subtle shifts in rumen microbial nucleic acid metabolism and subsequent transfer of nucleotide derivatives to the mammary gland, reinforcing the sensitivity of milk nucleotide composition to both microbial activity and host physiology. A pathway analysis further highlighted phosphoribosyl pyrophosphate (**PRPP**) as a critical intermediate connecting purine and pyrimidine metabolism. Phosphoribosyl pyrophosphate is a fundamental precursor in the de novo synthesis of both nucleobases (Martinussen et al., 2011), and its presence underscores the shared biochemical machinery underlying the observed seasonal patterns. The identification of PRPP as a network hub suggests that regulation at this metabolic crossroad could contribute to the seasonal redistribution of nucleotide derivatives in milk, linking environmental influences with core anabolic pathways in the cow. To further explore well-known biomarkers of nucleotide metabolism in cow milk, a semiquantitative analysis was performed on uric acid, allantoin, and BAIBA (Table 1). As expected, uric acid and allantoin, 2 established end-products of purine catabolism, did not differ significantly across seasons (*P* > 0.05), reflecting the controlled dietary conditions and the relatively stable rumen microbial protein synthesis ensured by the standardized corn silage-based TMR and similar DMI values. In this regard, the average dietary CP content (14.9% DM) recorded in this study represents a seasonal mean across the 4 sampling periods, rather than a targeted high-protein ration. Despite being lower than levels commonly recommended for high-producing cows in intensive systems (NASEM, 2021), this CP content was sufficient to sustain stable purine catabolite levels in milk, suggesting adequate microbial protein synthesis under the adopted feeding strategy. For example, a previous study by Rashid (1973) showed that the composition of the purine and pyrimidine derivatives of milk produced by protein-free cows and by normally fed cows was about the same. Also, the average ether extract content of the diets (4.1% DM), reflecting PDO-oriented feeding practices, was lower than levels commonly adopted in high-input systems (6%–7% DM); however, moderate fat inclusion is known to preserve rumen microbial activity and microbial crude protein synthesis (Palmonari et al., 2024), which may help explain the overall stability of milk purine end-products observed across seasons. In contrast, although similar DMI values could be observed (i.e., 26.28 kg/cow per day), we found that BAIBA, a catabolite of thymine and a marker of pyrimidine turnover, increased significantly in summer (*P* < 0.05), whereas purine catabolites such as uric acid and allantoin did not vary across seasons. Taken together, these results suggest that pyrimidine metabolism may be more sensitive to seasonal environmental influences than purine metabolism under standardized feeding conditions. As a general consideration, the absence of seasonal variation in milk allantoin and uric acid indicates that overall purine turnover and microbial protein synthesis remained relatively stable across seasons. The summer increase in BAIBA therefore likely reflects alterations in pyrimidine metabolism associated with seasonal environmental conditions rather than a direct effect of altered intake, considering that no significant DMI values were recorded during summer. Additionally, BAIBA can become part of β-alanine metabolism and valine, leucine, and isoleucine degradation (Kanehisa et al., 2014), thus indicating that it can function as an intermediate product in other parts of nitrogen metabolism. Elevated BAIBA levels in summer may reflect shifts in microbial activity or host metabolic adaptations to heat stress, as BAIBA is implicated in energy homeostasis and oxidative stress responses. Therefore, we postulate that in a controlled feeding context, only this specific pyrimidine-derived catabolite exhibits a seasonal variability, reinforcing its potential as a sensitive tracer of environmental stressors in dairy systems. However, milk nucleotide composition may not solely depend on rumen microbial turnover, but could also be influenced by mammary-specific processes (Gonzalez-Ronquillo et al., 2003; Rocchetti et al., 2022; Giagnoni et al., 2025). It is conceivable that nucleosides undergo exchange at the mammary level as part of cellular turnover and tissue metabolism. Although our study was not designed to investigate arterio-venous differences or interorgan fluxes, these mechanisms may represent an additional layer contributing to the nucleotide signature of milk and warrant further targeted investigation. It should also be acknowledged that part of the purine- and pyrimidine-related metabolites detected in milk may originate not only from rumen microbial turnover and mammary metabolism, but also from microbial fermentation processes occurring during corn silage ensiling, which can release nucleotide derivatives later entering the cow's metabolic network (Guo et al., 2023). The identification of purine- and pyrimidine-related metabolites in bovine milk provides new opportunities for studying nitrogen efficiency and rumen microbial dynamics. Traditionally, urinary allantoin and uric acid have been used to estimate microbial protein synthesis (Gonda and Lindberg, 1997; Shingfield and Offer, 1998; Gonzalez-Ronquillo et al., 2003), and recent advances include near-infrared spectroscopy prediction of urinary allantoin (Ribeiro et al., 2025). However, few studies have examined these metabolites directly in milk (Vlassa et al., 2021; Rocchetti et al., 2022; Fukuuchi et al., 2022; Giagnoni et al., 2025). The present work broadens this perspective, highlighting milk nucleotides as potential noninvasive indicators of microbial activity and nitrogen recycling. Future research should link milk-based nucleotide profiles with urinary purine excretion and rumen metagenomics, advancing predictive models of nitrogen use through multi-omic data integration (Lima et al., 2023). Our findings indicate that milk purine and pyrimidine metabolites carry season-dependent information even under a corn silage-based standardized diet. Multivariate models identified several nucleotide derivatives as seasonal discriminants, whereas only BAIBA showed a significant summer elevation. Overall, pyrimidine metabolism appeared particularly responsive to environmental variation, positioning BAIBA as a tracer of summer-related metabolic adaptation. To the best of our knowledge, this is the first systematic HRMS-based screening of purine and pyrimidine metabolites in bovine milk under controlled feeding and across seasons, underscoring their potential as biochemical indicators of environmental influences. It is recognized that the mammary gland possesses the metabolic capacity to synthesize and metabolize both purine and pyrimidine compounds, potentially contributing to the nucleotide profile of milk alongside rumen-derived precursors. Nevertheless, this study cannot determine whether these compounds primarily originate from rumen microbes or mammary metabolism, as it was designed as an observational screening. Future studies integrating splanchnic balance and isotope-tracing approaches are needed to clarify metabolic fluxes and establish the biological origin of these nucleotide-related compounds in milk.

**Figure 1:**
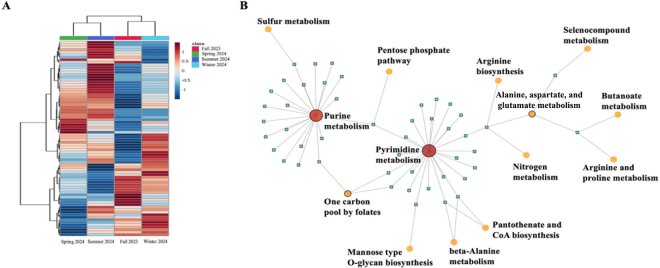
Overview of purine- and pyrimidine-related metabolites detected in cow milk across 4 seasons. (A) Heat map with hierarchical clustering of averaged metabolite intensities, showing unsupervised grouping of samples according to season. (B) Pathway-based network analysis linking annotated metabolites to major metabolic routes.

**Figure 2:**
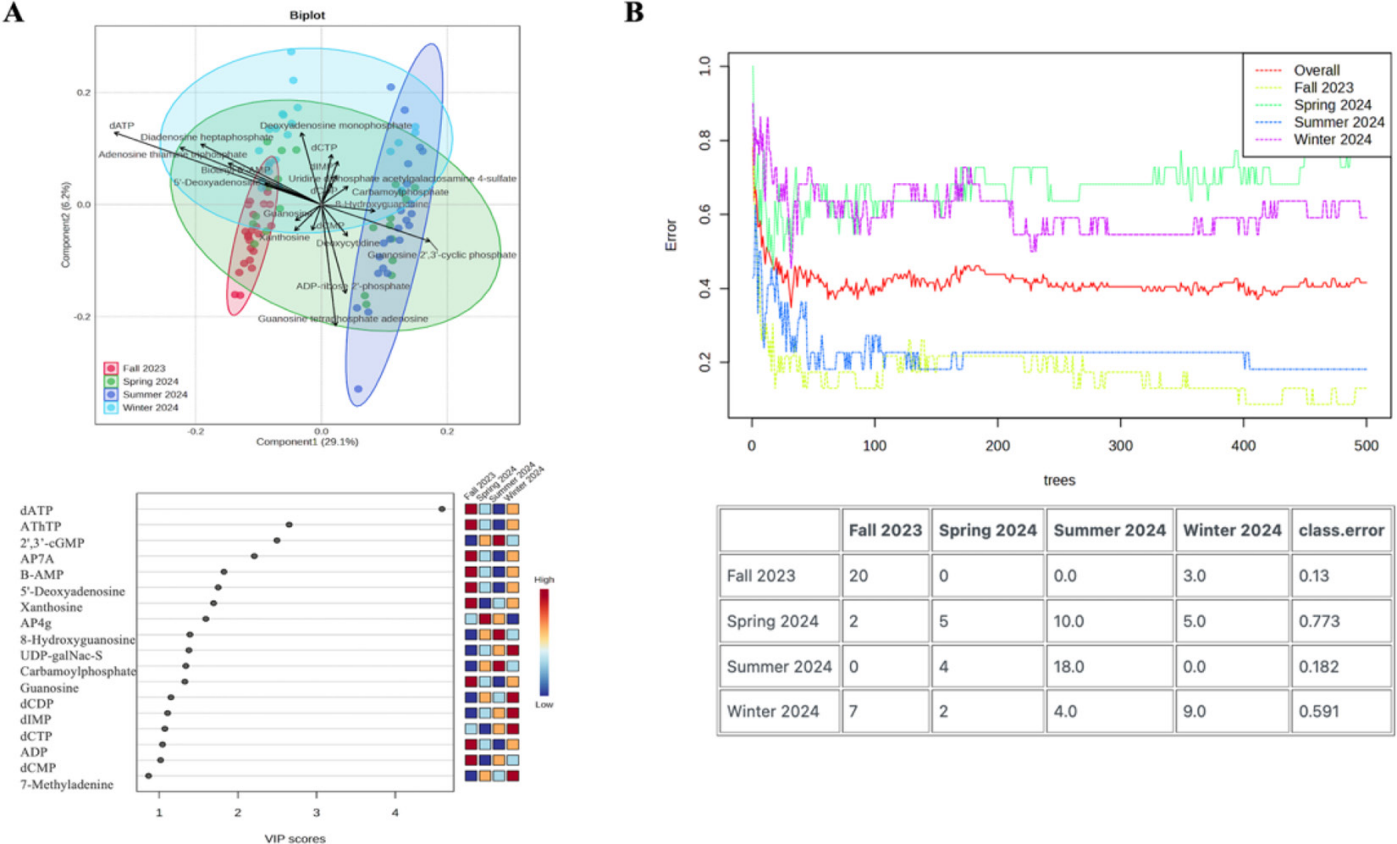
Multivariate and machine learning analyses of seasonal milk metabolome. (A) PLS-DA biplot illustrating the separation of milk samples according to the 4 seasons (top), and metabolites with the highest variable importance in projection (VIP >0.8), highlighting season-dependent trends (bottom). (B) Random forest classification performance, reporting the confusion matrix with error rates for each season, confirming the discriminant ability of season-specific metabolites. 2′,3′-cGMP = guanosine 2′,3′-cyclic phosphate; AP4g = guanosine tetraphosphate adenosine; AP7A = diadenosine heptaphosphate; AThTP = adenosine thiamine triphosphate; B-AMP = biotinyl-5′-AMP; dATP = deoxyadenosine triphosphate; dCDP = deoxycytidine diphosphate; dIMP = deoxyinosine monophosphate; dCTP = deoxycytidine triphosphate; dCMP = deoxycytidine monophosphate; UDP-galNAc-S = uridine diphosphate acetylgalactosamine 4-sulfate.

**Table 1:** Semiquantitative analysis of purine and pyrimidine degradation products considering their modifications across different seasons; results expressed as mean value ± SD

Metabolite (mg/L)	Pathway (*Bos taurus*)	Fall 2023	Winter 2024	Spring 2024	Summer 2024	Significance
BAIBA^1^	Pyrimidines	123.92 ± 16.27^a^	124.85 ± 18.38^a^	126.11 ± 15.36^a^	137.05 ± 19.37^b^	*P* = 0.049
Uric acid	Purines	2.96 ± 0.68	3.10 ± 0.45	3.00 ± 0.44	2.93 ± 0.43	*P* = 0.708
Allantoin	Purines	0.91 ± 0.14	0.91 ± 0.13	0.92 ± 0.10	0.93 ± 0.16	*P* = 0.917

a,b The presence of different superscript letters within a row indicates significant differences among milk samples (*P* < 0.05), as resulting from one-way ANOVA followed by Duncan's post hoc test.

1 BAIBA = β-aminoisobutyric acid.
